# Impermeable flexible liquid barrier film for encapsulation of DSSC metal electrodes

**DOI:** 10.1038/srep27422

**Published:** 2016-06-06

**Authors:** Junghee Yang, Misook Min, Yeoheung Yoon, Won Jung Kim, Sol Kim, Hyoyoung Lee

**Affiliations:** 1 Centre for Integrated Nanostructure Physics (CINAP), Institute of Basic Science (IBS), Department of Chemistry, and Department of Energy Science, Sungkyunkwan University, 2066 Seobu-ro, Jangan-gu, Suwon-si, Gyeonggi-do, Korea; 2 Hyundai Motor Group, Environment & Energy Research Team, 37, Cheoldobangmulgwan-ro, Uiwang-si, Gyeonggi-do, Korea

## Abstract

Encapsulation of electronic devices such as dye-sensitized solar cells (DSSCs) is prone to degradation under normal atmospheric conditions, even with hermetic barriers on the metal electrodes. Overcoming this problem is crucial to increasing DSSC lifetimes and making them commercially viable. Herein, we report a new impermeable flexible liquid barrier film using polyvinyl alcohol (PVA) and partially reduced graphene oxide (PrGO), which dramatically enhances the lifetime of Ag metal electrodes (typically used in DSSCs) immersed in a highly acidic iodolyte solution. The Ag metal electrode encapsulated by the PVA/PrGO film survived for over 500 hrs, superior to existing barriers of glass frits, epoxy resins and polymers. The PVA/PrGO film strongly adheres to the Ag metal surface, and the resulting PVA/PrGO/Ag electrode is stable even on a curved substrate, with a sheet resistance nearly independent of curvature. These results give new insight for the design of high-performance and solution-processable flexible liquid barrier films for a wide range of applications, in particular for the encapsulation of electronic devices with liquid electrolytes.

Flexible liquid and/or gas barrier films preventing the penetration of gas, moisture, and various kinds of solutions are essential for a number of industry applications^1^,^2^. For example, incorporation of dye-sensitized solar cells (DSSCs) into the sunroof and windows of cars has attracted strong interest as a green and eco-friendly energy source. Industrial researchers have been applying DSSC technology to various other kinds of electronic devices such as battery chargers and electric power suppliers^3^,^4^. However, so far commercially available DSSCs have not been successful due to the lack of a suitable liquid barrier film (LBF) that can protect the metal electrodes from the DSSCs strong acidic electrolyte solution.

Metal current collectors in DSSCs are generally prepared on conducting glass surfaces in much the same way as conventional photovoltaic cells^5^,^6^, in order to decrease the sheet resistance of the conductive glass substrate and to collect photo-excited carriers more efficiently^7^. Silver is the metal of choice because it has a low electrical resistance and low dark current. However, Ag metal grid lines in DSSCs need to be protected by a chemically stable and insulating coating film. Without the protection film, they will be easily corroded by the highly active I^−^/I_3_^−^ redox electrolyte^7^,^8^. Contact between the Ag metal grids and the electrolyte easily leads to increased dark current through charge recombination^9^,^10^. Thus, a major challenge is finding a stable and impermeable LBF that is eco-friendly and (preferably) transparent to encapsulate metal electrodes immersed in DSSC electrolyte solution. The LBF should be chemically inert, and its fabrication should be compatible with solution processes.

In previous works, many researchers studied various alternative coating layers such as glass frit, epoxy resin, zinc borosilicate, and UV curing^11^,^12^,^13^,^14^. Some groups tried to protect the Ag metal grids from the redox active electrolyte by a Surlyn film sheet lamination known as a glass ceramic overcoat layer, while others applied anti-corrosion coating agents on the Ag grids^15^. However, existing protective layers on Ag electrodes are rigid and easily broken. Furthermore, existing LBFs do not even prevent the infiltration of liquid electrolytes from the DSSC module. Therefore, the development of flexible LBFs as an insulating coating layer on metal electrodes is urgently needed.

Recently, two-dimensional (2D) graphene oxide (GO) has been introduced as an adhesive gas barrier film (GBF) on a large scale^16^,^17^,^18^,^19^,^20^,^21^. However, even though it has good adhesion to Ag metal electrodes, the use of GO by itself as a LBF is insufficient because it does not prevent leaking of the electrolyte onto the metal electrodes. Therefore GO has only been used as a gas barrier so far. Hybrid materials like graphene-oxide-based polymer nano-composites (GO–polyvinyl alcohol (PVA) film) have also been introduced as hydrogen gas barriers^17^,^22^,^23^. Polymer nanocomposites with graphene have been studied to obtain polymeric gas barrier materials, taking advantage of the gas barrier properties of graphene *via* its incorporation into a polymer matrix and the surface coating of the polymeric substrate. Previous studies have also focused on the fabrication of graphene-based polymer sheets for oxygen, carbon dioxide, water vapor, and nitrogen GBFs^1^,^22^.

In contrast to gas, however, liquid tends to pass easily through barrier films due to swelling when the film is in contact with the electrolyte solution^24^. At the interface, liquid can easily permeate the film formation layer so it is very difficult to protect against polar liquid solvents using conventional GBFs. Thus GO-based polymer films cannot be used since the hydrophilic GO either absorbs polar solvents or disperses into them, resulting in permeation of the electrolyte solution to the metal electrodes^22^,^24^. An additional problem with GO arises because although good adhesion to metal is obtained, oxygen functional groups in GO readily oxidize the Ag metal surface leading to dark spots. Unfortunately, to date there is no report of an LBF which can completely prevent the leaking of a liquid polar electrolyte onto submerged metal electrodes including DSSC electrodes.

In the search for novel LBFs, the concept of a double-layered structure consisting of a hydrophilic polymer film facing the polar electrolyte solvent and a hydrophobic film attached to the metal electrodes may solve the problem of the polar solvent leaking onto the metal electrodes. It is expected that a stable hydrophilic polymer film deposited on top of a hydrophobic film^25^, that is, a vertically developed nano-pored hydrophilic film with a hydrophobic surface structure at the bottom layer, can prevent the polar solvent from penetrating the double-layered film due to the strong repulsion of the polar solvent at the hydrophobic nano-scaled interface^26^.

PVA is a common organic polymer for barrier film applications which may be a good choice for the hydrophilic film layer^1^,^27^. From previous reports, PVA is known as an excellent GBF for O_2_, N_2_, H_2_, He, Ar, and carbonic acid gases^1^. For example, PVA has an oxygen transmission rate (OTR) so low that it is often used as a benchmark for the amount of O_2_ (g) that goes through a substance^22^. PVA is also chemically stable in organic solvents^28^. However, PVA films do allow penetration of moisture and NH_3_ gas^1^. On the other hand, reduced graphene oxide (rGO) prepared by *in-situ* thermal reduction of GO films is a good choice for the hydrophobic film directly attached to the Ag metal electrode. It is expected that strong π-π electron interactions between rGO nanosheets reduce the interlayer distance of rGO films, and their hydrophobic property effectively repels polar electrolyte solvents^22^.

In this paper we report a novel LBF consisting of double-layered hydrophilic PVA and hydrophobic partially reduced graphene oxide (PrGO) hybrid films for the protection of Ag metal electrodes from strong oxidizing polar liquid electrolyte solutions. As mentioned earlier, we chose PrGO nanosheets for the hydrophobic film attached to the metal electrode, and PVA for the hydrophilic film that is stable in polar organic solvents. The PrGO film is thermally reduced from GO and maintains good adhesion on the Ag metal electrode. Furthermore, strong interfacial interactions formed by ester chemical bonding between PVA and PrGO films result in high mechanical stability^29^. It has been previously reported that the tensile strength and Young’s modulus of PVA/PrGO composites are vastly improved^30^,^31^. With these advantages, double-layered PVA/PrGO films can be flexibly applied to winding-edge surfaces, and are expected to effectively prevent the penetration of polar electrolyte solvent onto even thick Ag electrodes. An encapsulation consisting of uniform and continuous double-layered PVA/PrGO thin films significantly extends the lifetime of Ag electrodes immersed in iodolyte solution typically used for DSSCs to over 500 hrs, which is superior to existing barriers of glass frit, epoxy resin and polymer. These results provide new insight for the design of high-performance and solution-processable transparent LBFs for the encapsulation of a wide range of electronic de**v**ices that use liquid electrolytes.

## Results and Discussion

### Counter electrode of parallel grid DSSC protected by PVA/PrGO hybrid film

**Figure 1:**
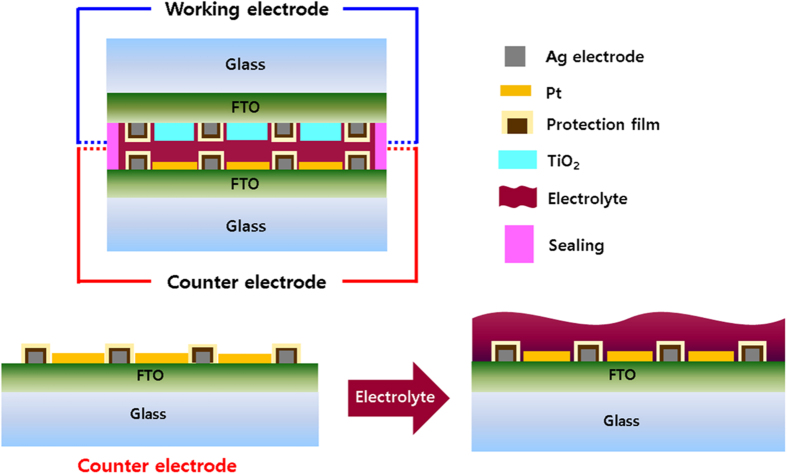
DSSC structure of parallel grid. We fabricated barrier films against electrolyte solution. Corrosion protection from using PVA/PrGO barrier film in the counter electrode.

A parallel-type grid module is widely used for DSSCs (Fig. 1a and Supplementary Fig. S1a)^7^,^8^, and Ag is often used as the electrode due to its high conductivity and low electrical resistance^11^. The counter-electrode consists of both Ag and Pt, used as a catalyst, and is fabricated by a printing technique (Fig. 1 and Supplementary Fig. S1a)^8^. Scanning electron microscopy (SEM) images of the Ag grid lines are shown in Supplementary Fig. S1b. However, as mentioned earlier, Ag degradation occurs due to the penetration of a redox active and corrosive electrolyte solution, necessitating the development of LBFs to prevent leaking of the electrolyte solution onto the metal electrodes. An illustration of the fabrication procedure is shown in Fig. 1. To fabricate PVA/PrGO hybrid films as protective passivation layers, first an aqueous solution containing GO was applied to the Ag electrode at 70 °C using a spray coating technique to give a uniform GO film. Next, *in-situ* thermal treatment at 300 °C with 4% H_2_/Ar (g) under high vacuum condition (~10^−7^ Torr) for 5 hrs reduced the GO, forming a PrGO film with both carboxylic acid and hydroxyl functional groups. Finally the PrGO nanosheet films were coated with PVA solution. The resulting double-layered films were dried at 80 °C for 24 h and then heated 100 °C for 2 h. Schematic structures and SEM images of GO (Supplementary Fig. S2a,d), PrGO (Supplementary Fig. S2b,e), and PVA/PrGO hybrid films (Supplementary Fig. S2c,f) on the Ag electrode are shown. Throughout partial hydrogen and chemical bonding, SEM cross-section micrographs showed that the PVA/PrGO matrix coated the Ag electrode^31^. The PVA film on the PrGO film is a thin, uniform layer without apparent microstructural defects such as micropores.

**Figure 2:**
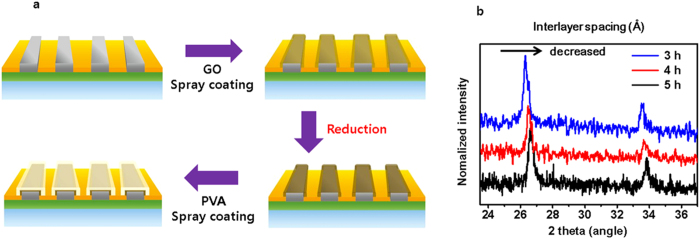
Fabrication of PVA/PrGO hybrid film. (**a**) In counter electrode, schematic illustration of the fabrication process of the PVA/PrGO hybrid film on the metal electrode/glass. GO suspension is spray-coated on Ag electrode, reduced at 300 °C under 4% H_2_/Ar to become PrGO, and finally 3 wt% PVA is spray-coated on the PrGO layer/Ag electrode. (**b**) A XRD data of thermally treated PrGO on time. (3 h, 4 h and 5 h).

Using cross-sectional SEM we measured the GO film thickness to be 0.15–0.16 μm, and the PVA film thickness to be 5.6 μm. The overall thickness of the PVA/PrGO double-layered film on the Ag electrode is therefore about 5.7–6.0 μm (Supplementary Fig. S2f). For chemical and surface characterization of the insulating films, Raman mapping microscopy, X-ray photoelectron spectroscopy (XPS), and X-ray diffraction (XRD) were performed (Supplementary Fig. S3). Raman mapping spectra of GO, PrGO, and PVA/PrGO hybrid films are shown in Supplementary Fig. S3a–d. The typical Raman spectrum of a GO film, as expected, displays a prominent G peak at 1350 cm^−1^ and a D peak at 1670 cm^−1^ due to sp^2^–hybridized C-C bonds. The Raman spectrum of the PrGO film also contains D, G, and 2D peaks at 1400 cm^−1^, 1710 cm^−1^, and 2650 cm^−1^, respectively^16^,^32^. PVA/PrGO hybrid films show a 2D peak at 2810 cm^−1^ (compared with the 2D peak of graphene at 2700 cm^−1^), and 2D peaks of the pointed PVA films generally appear around 2800 cm^−1^. Therefore, through Raman mapping (100 × 100 μm) we can see that the whole area is well coated by the film. Next, XPS was employed to verify the chemical composition of the GO and PrGO films (Supplementary Fig. S3e). The C1s spectrum of the GO film contains multiple peaks corresponding to different oxidation states of carbon: C-C (284.6 eV), C-O (286.3 eV), and C=O (288.1 eV). After thermal reduction, the C-O peak of PrGO decreased in intensity compared to GO, and peaks are slightly shifted to lower binding energy: C-O (286.1 eV), and C=O (287.9 eV). Several small oxygen groups remain, consistent with previous results from thermally reduced PrGO films (Supplementary Fig. S4). The XRD pattern for the PrGO film shows a diffraction peak at 26.50° while the PVA shows a peak at about 20°. The PVA/PrGO hybrid barrier films show diffraction peaks at 19.76° and 26.50° confirming the presence of both PVA and PrGO, respectively (Supplementary Fig. S3f). Interestingly, as the reduction time increased from 3 hrs to 5 hrs, the interlayer spacing obtained from XRD decreased and the peaks became sharper, eventually giving a graphite-like interlayer distance (Fig. 2b and Supplementary Fig. S5). As the reduction time increased, the graphitization of PrGO films increased and the number of defect sites decreased, leading to a high crystallinity of PrGO films (Fig. 2b)^24^.

To test for adhesion, the barrier film was stripped away by a strong external force. In the process, parts of the electrode were removed along with the barrier films, indicating that the PrGO films were strongly bonded to the Ag electrode as confirmed by OM and SEM images (Supplementary Fig. S6). In previous studies, Ag NWs were uniformly covered by rGO films^33^. Ag NPs form strong covalent bonds to rGO and PrGO. Their chemical interaction can be explained in detail using XPS. In addition, it was reported that the rGO films on Ag NWs hybrid electrode show high optical transmittance, low sheet resistance, enhanced thermal oxidation and chemical stability^34^. Ag NWs with the help of the rGO barrier films can survive in harsh, adverse conditions for a long time. Surprisingly, even as mentioned earlier, the thermal reduction of GO into PrGO results in strong adhesion to the Ag electrode (Supplementary Fig. S6). When we attempted to remove the PVA/PrGO hybrid films from the substrate in which the Ag grid electrodes are embedded, the film did not detach from the Ag electrode as it was lifted from the glass substrate, which means that the PrGO film was strongly bonded to the Ag (Supplementary Video 1). It is quite interesting to ask why PrGO that is thermally reduced from GO can strongly bind to Ag. The bonding may be due to carboxylic acid and hydroxyl functional groups in the PrGO film, like sorbitol, which is known as an adhesive agent^33^. To test this hypothesis, we controlled the number of carboxylic acid and hydroxyl functional groups by varying the reduction temperature from 300 to 500 °C (Supplementary Fig. S7)^35^. At higher temperatures, the number of carboxylic acid and hydroxyl functional groups is reduced, and the adhesion strength of the PrGO film to Ag electrode should decrease. In fact, films prepared at 500 °C (Supplementary Fig. S7b) detached more easily than those prepared at 400 °C (Supplementary Fig. S7c) and then 300 °C (Supplementary Fig. S7d) in order. In other words, films prepared at 300 °C in vacuum for 5 hrs are the most strongly bonded to the Ag electrodes among those prepared at 300, 400 and 500 °C. On the other hand, PrGO films prepared at a temperature lower than 200 °C are not suitable for LBFs since too many oxygen functional groups lead to a hydrophilic, rather than a hydrophobic, surface. In addition, PVA placed on a PrGO film chemically attaches to PrGO *via* ester chemical bonding between the hydroxyl group of PVA and carboxyl group of PrGO at 300 °C. Fourier transform infrared spectra (FT-IR) shows a new and high intense C=O stretching peak of the ester functional group at 1738 cm^−1^, indicating that the layer-structured PVA/PrGO film is strongly chemically bonded (Supplementary Fig. S8)^36^. Thus, due to the strong binding of PrGO film to the metal electrodes and chemical ester bonding between PVA and PrGO, the double-layered and chemically bonded PVA/PrGO hybrid film is well adhered on the winding and even the rectangular top edge surface of the Ag electrode^28^,^31^.

### A long- term stability test of PVA/PrGO/Ag in liquid electrolyte

**Figure 3:**
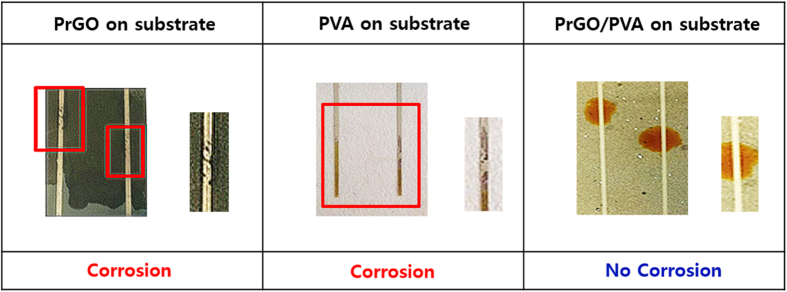
Long-term corrosion tests after immersion in electrolyte solution for 100 hrs. (**a**) PrGO film on Ag. (**b**) PVA coating on Ag. (**c**) PVA/PrGO hybrid film on Ag. Only PVA/PrGO film is stable in iodolyte electrolyte solution for a long-term corrosion test.

A long-term stability test of the newly passivated metal electrodes was performed by immersing three different samples (PrGO/Ag, PVA/Ag, and PVA/PrGO/Ag) into corrosive electrolyte solution (Fig. 3 and Supplementary Fig. S9). After immersion for 100 hrs, black spots were observed on both PrGO/Ag and PVA/Ag electrodes due to penetration of the electrolyte solution onto the metal electrode (Fig. 3a,b), while black spots for the PVA/PrGO/Ag electrode was not observed at all. Among all three samples, only the PVA/PrGO/Ag electrode was not corroded (Fig. 3c). Black spots were still not observed on the PVA/PrGO/Ag electrode after immersion from 200 to 500 hrs, meaning that no corrosion occurred in the strong iodine electrolyte solution (Supplementary Fig. S9). After immersion over 500 h, the Ag was etched a little by the electrolyte, but the PVA/PrGO/Ag electrode endured. Thus, we have demonstrated that the PVA/PrGO hybrid films can be used to protect Ag electrodes for over 500 hrs from the iodolyte DSSC solution. After immersed 500 hrs, it checked images about the number of GO spray coating and PVA coating in shown Supplementary Fig. S9f. Ag electrode have a good stability that while the number of coating increased. And Ag electrode leads to corrosion from edge. Between glass substrate and PrGO have a low adhesion. By this cause, PVA/PrGO/Ag sample start to show black spot after 500 hr.

**Figure 4:**
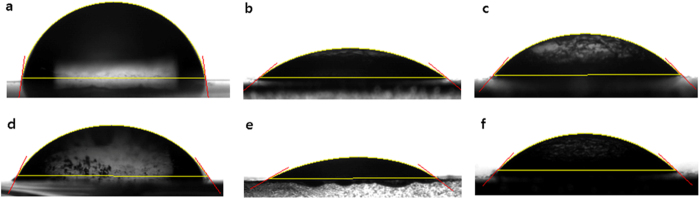
Characterization of swelling by using Water contact angles. (**a–c**) Before the electrolyte test and (**d–f**) after immersion for 5 hrs in electrolyte solution. (**a,d**) PrGO on Ag electrode/glass. (**b,e**) PVA on Ag electrode/glass. (**c,f**) PVA/PrGO hybrid film on Ag electrode/glass.

To learn about the surface properties of the LBF, water contact angles (WCAs) were measured after immersion in iodolyte solution for 5 hrs. In particular, the WCA is an indicator of the wettability and surface roughness^37^,^38^. After immersion, the WCA for the PrGO/Ag electrode sharply decreased from 81.97° to 60.11° and the WCA for the PVA/Ag electrode decreased from 38.97° to 27.63° (Fig. 4). However, WCAs of the PVA/PrGO hybrid films slightly increased from 45.19° to 46.07° or showed almost no change, which means that the iodolyte solution did not modify the surface properties of the LBF. The durability of the film surface during immersion may hinder penetration of the electrolyte solution into the LBF. This theory also explains why defect sites of over-coating layers cause a swelling effect^39^,^40^. When the film is wet, surface wrinkling appears and an instability-driven pattern is generated. From these WCA measurements we can conclude that the surface structures of PVA and PrGO films are modified by the electrolyte solution to allow the solution to penetrate, while those of PrGO/PVA hybrid films are unchanged, forming a highly stable and effective LBF. The synergy effect of PVA/PrGO hybrid films gives rise to its excellent barrier property. PVA is stable against the electrolyte solution, and the remaining oxygen functional groups of partially reduced hydrophobic PrGO serve as a good linker to the Ag metal surface and simultaneously to the PVA film. As the thickness of spray-coated PrGO films increased, the hydrophobicity of the PrGO film also increased^41^, as confirmed by the order of WCAs (c) 66.90°, (d) 70.59°, (e) 75.37°, and (f) 93.15° (Supplementary Fig. S10), whereas WCAs of bare and GO films indicate a high hydrophilic property (a) 31.85° and (b) 38.05°, respectively (Supplementary Fig. S10a,b).

**Figure 5:**
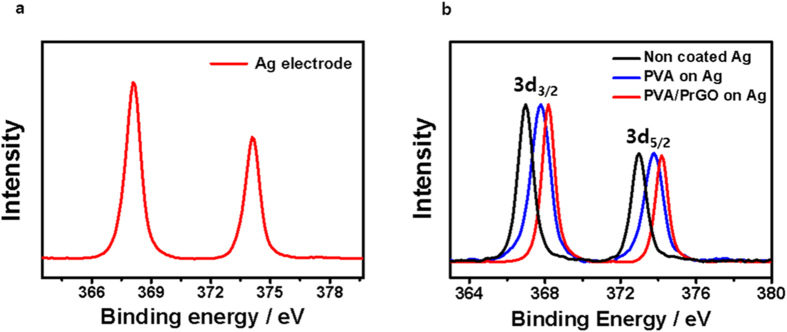
Chemical analysis technique. (**a**) XPS Ag 3d_5/2_ spectrum of non-coated Ag electrode before electrolyte treatment. (**b**) XPS Ag 3d_5/2_ spectra of bare Ag, PVA/Ag, and PVA/PrGO/Ag electrodes after electrolyte treatment.

In addition, to confirm that the electrolyte does not permeate into the PVA/PrGO hybrid barrier films, the Ag electrodes were immersed in iodolyte solution for 100 hrs, and XPS analysis of the Ag metal surface was performed to look for evidence of chemical bonding between Ag and iodide. The XPS binding energy of Ag 3d_5/2_ for Ag metal is 368.2 eV (Fig. 5a), and the Ag 3d_5/2_ binding energy of the PVA/PrGO/Ag electrode is also 368.2 eV, indicating that no iodine contamination is present on the Ag metal surface of the PVA/PrGO/Ag electrode. On the other hand, PVA-coated Ag and bare Ag electrodes show distinct peak shifts due to Ag-I bonding. The peak shift of bare Ag is larger than that of the PVA/Ag electrode, indicating greater iodine contamination (Fig. 5b). Therefore, based on XPS analysis we can conclude that only PVA/P-PrGO film can be used as an LBF for protection of Ag electrodes in the iodolyte solution.

**Figure 6:**
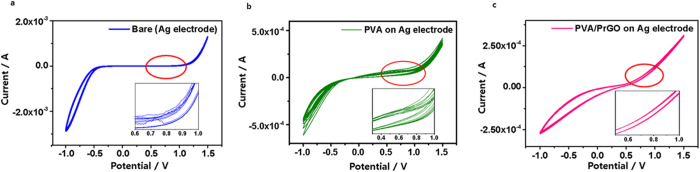
Electrochemical stability test in electrolyte. (**a**) Cyclic voltammetry of not coated Ag electrode (bare). (**b**) After PVA coating, and (**c**) After PVA/PrGO coating. Of these three PVA/PrGO has the highest stability after repeated cycles.

To measure long-term device performance an electrochemical stability test was also performed^42^,^43^. A three-electrode flow cell was constructed and nanocomposite-coated Ag was used as the working electrode. The reference electrode and counter-electrode were gold-coated gold wire (Au/AuCl) and platinum foil, respectively. Unless otherwise stated, all measurements were performed at a scan rate of 0.1 V/s. Figure 6 shows the cyclic-voltammograms (C-V) for all Ag electrodes^44^ recorded in 50 mM iodide-based redox electrolyte solution (Iodolyte AN-50). Redox C-V curves of all Ag electrodes show that the electrochemical behavior depends on the size of the counter ions present in the electrolyte, in order to neutralize charges formed through the oxidation-reduction reactions. Generally as the potential increases, the current increases. C-V curves of the bare Ag electrode begin to flatten at around 0.8 V after 10 cycles (Fig. 6a), while C-V curves of the PVA/Ag electrode change at around 0.8 V after 50 cycles (Fig. 6b). However, redox C-V curves of the PVA/PrGO/Ag electrode were stable even after 100 cycles, which means that PVA/PrGO/Ag electrodes are suitable for long-term stable devices (Fig. 6c).

The remarkable barrier property of the PVA/PrGO/Ag electrode was also confirmed using energy dispersive X-ray (EDX) spectroscopy. EDX data from a PVA/Ag film on a glass substrate is displayed in Supplementary Fig. S11–1: (top) cross-sectional image in SEM, and (bottom) elemental composition on the surface of the Ag metal electrode. Table of EDX Supplementary Fig. S11–1 showed 3 wt% iodine from iodolyte and 7 wt% nitrogen from acetonitrile, meaning that the PVA film did not prevent leaking of the iodine electrolyte acetonitrile solution. On the other hand, the PVA/PrGO hybrid film proved to be much more stable than the PVA film^17^,^18^,^19^,^20^. Table of EDX (Supplementary Fig. S11–2) did not show evidence of iodine or nitrogen at all, meaning that the electrolyte solution did not permeate through the LBF. To test for applicability to flexible devices, the PVA/PrGO/Ag electrodes were wrapped on a curved bar (Supplementary Fig. S12). After the Ag electrodes were immersed in iodolyte solution for 100 hrs, the sheet resistance of the bare Ag, PrGO/Ag and PVA/Ag shows the high value (~10^2~3^ Ω) or sometimes no current since the Ag electrode is disconnected by electrolyte’s corrosion. The sheet resistance of curved and straight PVA/PrGO/Ag electrodes remained the same (2~4 Ω), while that of bare Ag and PVA-coated Ag electrodes changed dramatically. This results showing that PVA/PrGO/Ag electrodes can be applied to curved or flexible devices^45^,^46^.

## Conclusion

For the first time, a novel LBF consisting of PVA/PrGO hybrid layers on an Ag electrode has been successfully fabricated by spray coating, a very simple and inexpensive method. As designed, partially reduced hydrophobic PrGO strongly adheres to the Ag metal surface, and hydroxyl groups in PVA strongly bond to oxygen functional groups of PrGO through ester chemical bonding, leading to a highly stable double-layered film that is strongly bonded to the Ag metal. The newly obtained 5-μm-thick LBF showed a dramatically enhanced ability to prevent the permeation of an iodolyte solution onto Ag metal electrodes resulting in long-term stability of over 500 hrs, and may enable the development of commercially successful DSSCs using a strong electrolyte solution. Our concept of a double-layered structure can guide the search for new encapsulation methods for the protection of metal electrodes from liquid solutions. These results give new insight for the design of solution-processable transparent flexible LBFs for a wide range of applications, especially in the encapsulation of electronic devices that use liquid electrolytes.

## Method

### Silver metal grids

Silver is the metal of choice for grid line application in solar cells because it has a high conductivity and low dark current. The Ag electrode/FTO/Pt/Glass stack used as a transparent electrode was provided by Hyundai Motor Group. Silver grid lines (dimension, length ∙ width ∙ height: 94 mm ∙ 1.0 mm ∙ 0.6 mm) were printed on conductive glass substrate using screen printable sliver paste. The sizes 200 mm × 100 mm designed screen that cover the whole surface of the metal grids and cured at 500 °C for 2 hrs.

### Preparation of the GO Solution

By Hummers method, GO (graphene oxide) was synthesized from graphite powder (Bay carbon, SP-2) using KMnO_4_ as an oxidizer. The synthesized GO was characterized as described in a previous report. 2.5 mg/mL GO in deionized (DI) water was mixed together and sonicated for 24 hr. Water-dispersed GO solution was centrifuged at 200 rpm for 1 hr and then left standing for 1 hr.

### Fabrication of PVA/PrGO insulating hybrid film

Supernatant GO solution was printed on Ag electrode lines, following the bar coating technique. The GO-coated Ag electrode lines were then dried in a vacuum desiccator overnight. After drying, partially reduced graphene oxide (PrGO) was formed by 300 °C heat treatment for 5 hrs under 4% H_2_/Ar (g) using a high-temperature furnace (Atech System, Korea), and slowly cooled to room temperature. To fabricate the PVA/PrGO hybrid films, 3 wt% PVA solution in water was deposited on top of the PrGO film by spray coating. Finally the resulting the PVA/PrGO hybrid films were dried at 80 °C for 24 h and then heated 100 °C for 2 h in vacuum.

### Characterization

WCA measurements were performed to determine the wettability of film surfaces using an SEO Phoenix 300 microscope. Raman spectra were measured using a WITEC Alpha 300 spectrometer with a 532 nm excitation wavelength. Sample morphology was characterized by SEM (FEI NOVA Nano SEM 200 or JEOL JSM-7600 F) and optical microscopy (OM, Bimeince MIC). Bonding between the PrGO and PVA films was measured by Fourier transform infrared spectroscopy (Bruker IFS-66/S). X-Ray Diffraction (XRD) measurements were performed to measure interlayer spacing using a Bruker D8 ADVANCE diffractometer. X-ray Photoemission spectroscopy (XPS) was performed to measure chemical composition and surface bonding states using a Thermo Scientific K-Alpha spectrometer, equipped with an Mg/Al X-ray source. Cyclic voltammetry (CV) was performed to measure the long term stability of LBF-coated Ag electrodes immersed in strong electrolyte solution using a CHI Electrochemical Analyzer 660A. A three-electrode electrochemical cell consisted of a Pt counter electrode, an Ag/AgCl reference electrode and PVA, PrGO, PVA/PrGO on Ag working electrode.

## Additional Information

**How to cite this article**: Yang, J. *et al.* Impermeable flexible liquid barrier film for encapsulation of DSSC metal electrodes. *Sci. Rep.* **6**, 27422; doi: 10.1038/srep27422 (2016).

## Supplementary Material

Supplementary Information

Supplementary Video 1

## References

[b1] Yoo B. M., Shin H. J., Yoon H. W. & Park H. B. Graphene and Graphene Oxide and their uses in Barrier Polymers. J. Appl. Polym. Sci. 131, 39628–39650 (2014).

[b2] Guilbert S., Gontard N. & Cuq B. Technology and Applications of Edible Protective Films. Packag. Technol. Sci. 8, 339–346 (1995).

[b3] Späth M. *et al.* Reproducible Manufacturing of Dye-Sensitized Solar Cells on a Semi-automated Baseline. Prog. Photovolt: Res. Appl. 11, 207–220 (2003).

[b4] Lewis J. S. & Weaver M. S. Thin-Film Permeation-Barrier Technology for Flexible Organic Light-Emitting Devices. IEEE J. Sel. Topics Quantum Electron. 10, 45–57 (2004).

[b5] Dang X. *et al.* Virus-Templated Self-Assembled Single-Walled Carbon Nanotubes for Highly Efficient Electron Collection in Photovoltaic Devices. Nat. Nanotechnol. 6, 377–384 (2011). 21516089 10.1038/nnano.2011.50

[b6] Tan Y., Song Y. & Zheng Q. Hydrogen bonding-driven rheological modulation of chemically reduced graphene oxide/poly(vinyl alcohol) suspensions and its application in electrospinning. Nanoscale 4, 6997–7005 (2012). 23037898 10.1039/c2nr32160b

[b7] Ramasamy E., Lee W. J., Lee D. Y. & Song J. S. Portable, Parallel Grid Dye-Sensitized Solar Cell Module Prepared by Screen Printing. J. Power Sources 165, 446–449 (2007).

[b8] Takeda Y., Kato N. & Toyoda T. Advances in Monolithic Series-Interconnected Solar-Cell Development. SPIE News room 1, 2009–2011 (2009).

[b9] Wang M. *et al.* An Organic Redox Electrolyte to rival Triiodide/Iodide in Dye-Sensitized Solar Cells. Nat. Chem. 2, 385–389 (2010). 20414239 10.1038/nchem.610

[b10] Reynolds G. J., Watson T. M., Williams G. & Worsley D. Corrosion Resistance of Metallic Substrates for the Fabrication Dye-Sensitized Solar Cells. ECS Transactions 33, 129–138 (2011).

[b11] Lee W. J., Ramasamy E., Lee D. Y. & Song J. S. Glass Frit Overcoated Silver Grid Lines for Nano-Crystalline Dye Sensitized Solar Cells. J. Photochem. Photobiol. A: Chem. 183, 133–137 (2006).

[b12] Wang X., Song L., Pornwannchai W., Hu Y. & Kandola B. The Effect of Graphene Presence in Flame Retarded Epoxy Resin Matrix on the Mechanical and Flammability Properties of Glass Fiber-Reinforced Composites. Composites: Part A 53, 88–96 (2013).

[b13] Yeon D.-H., Lee E.-Y., Kim K.-G., Park N.-G. & Cho Y.-S. Zinc Borosilicate Thick Films as a Ag-Protective Layer for Dye-Sensitized Solar Cells. J. Korean Ceram. Soc. 46, 313–316 (2009).

[b14] Toker R. D., Kayaman-Apohan N. & Kahraman M. V. UV-Curable Nano-Silver Containing Polyurethane Based Organic–Inorganic Hybrid Coatings. Prog. Org. Coat. 76, 1243–1250 (2013).

[b15] Shimomura A., Horikawa H., Shimomura J., Hasegawa Y. & Ogihara T. Preparation and Characterization of Silica Films on Stainless Steels Substrate by Dip Coating with PDMS/PHPS Solution. Trans. Mater. Res. Soc. Jpn. 37, 27–30 (2012).

[b16] Moon I. K. *et al.* 2D Graphene Oxide Nanosheets as an Adhesive Over-Coating Layer for Flexible Transparent Conductive Electrodes. Sci. Rep. 3, 1112–1118 (2013).

[b17] Cui Y., Kundalwal S. & Kumar S. Gas Barrier Performance of Graphene/Polymer Nanocomposites. Carbon 98, 313–333 (2016).

[b18] Berry V. Impermeability of Graphene and Its Applications. Carbon 62, 1–10 (2013).

[b19] Xiang C. *et al.* Functionalized Low Defect Graphene Nanoribbons and Polyurethane Composite Film for Improved Gas Barrier and Mechanical Performances. ACS nano 7, 10380–10386 (2013). 24102568 10.1021/nn404843n

[b20] Hu J. *et al.* A Review on the use of Graphene as a Protective Coating against Corrosion. Ann. J. Materials. Sci. Eng. 1, 16–31 (2014).

[b21] Zhao Y. *et al.* Highly Impermeable and Transparent Graphene as an Ultra-Thin Protection Barrier for Ag Thin Films. J. Mater. Chem. C 1, 4956–4961 (2013).

[b22] Layek R. K., Das A. K., Park M. U., Kim N. H. & Lee J. H. Layer-Structured Graphene Oxide/Polyvinyl Alcohol Nanocomposites: Dramatic Enhancement of Hydrogen Gas Barrier Properties. J. Mater. Chem. A 2, 12158–12161 (2014).

[b23] Chen J.-T. *et al.* Enhancing Polymer/Graphene Oxide Gas Barrier Film Properties by Introducing New Crystals. Carbon 75, 443–451 (2014).

[b24] Su Y. *et al.* Impermeable Barrier Films and Protective Coatings Based on Reduced Graphene Oxide. Nat. Commun. 5, 4843–4847 (2014). 25208890 10.1038/ncomms5843

[b25] Nine M. J., Cole M. A., Johnson L., Tran D. N. & Losic D. Robust Superhydrophobic Graphene-Based Composite Coatings with Self-Cleaning and Corrosion Barrier Properties. ACS Appl. Mater. Interfaces 7, 28482–28493 (2015). 26632960 10.1021/acsami.5b09611

[b26] Dong Y., Ma L. & Zhou Q. Effect of the Incorporation of Montmorillonite-Layered Double Hydroxide Nanoclays on the Corrosion Protection of Epoxy Coatings. J. Coat. Technol. Res. 10, 909–921 (2013).

[b27] Suk H. J. *et al.* Modified Polyvinyl Alcohol Layer with Hydrophobic Surface for the Passivation of Pentacene Thin-Film Transistor. J. Nanosci. Nanotechnol. 12, 3214–3218 (2012). 22849091 10.1166/jnn.2012.5643

[b28] Liu H.-W., Hu S.-H., Chen Y.-W. & Chen S.-Y. Characterization and Drug Release Behavior of Highly Responsive Chip-Like Electrically Modulated Reduced Graphene Oxide–Poly (vinyl alcohol) Membranes. J. Mater. Chem. 22, 17311–17320 (2012).

[b29] Hwang S.-H., Kang D., Ruoff R. S., Shin H. S. & Park Y.-B. Poly (vinyl alcohol) Reinforced and Toughened with Poly (dopamine)-Treated Graphene Oxide, and its Use for Humidity Sensing. ACS nano 8, 6739–6747 (2014). 24911396 10.1021/nn500504s

[b30] Feng H., Li Y. & Li J. Strong Reduced Graphene Oxide–Polymer Composites: Hydrogels and Wires. RSC Adv. 2, 6988–6993 (2012).

[b31] Li J., Shao L., Zhou X. & Wang Y. Fabrication of High Strength PVA/rGO Composite Fibers by Gel Spinning. RSC Adv. 4, 43612–43618 (2014).

[b32] Shojaee S. A., Zandiatashbar A., Koratkar N. & Lucca D. A. Raman Spectroscopic Imaging of Graphene Dispersion in Polymer Composites. Carbon 62, 510–513 (2013).

[b33] Ahn Y., Jeong Y. & Lee Y. Improved Thermal Oxidation Stability of Solution-Processable Silver Nanowire Transparent Electrode by Reduced Graphene Oxide. ACS Appl. Mater. Interfaces 4, 6410–6414 (2012). 23206541 10.1021/am301913w

[b34] Liu Y., Chang Q. & Huang L. Transparent, Flexible Conducting Graphene Hybrid Films with a Subpercolating Network of Silver Nanowires. J. Mater. Chem. C 1, 2970–2974 (2013).

[b35] Moon I. K., Lee J., Ruoff R. S. & Lee H. Reduced Graphene Oxide by Chemical Graphitization. Nat. Commun. 1, 73 (2010). 20865806 10.1038/ncomms1067

[b36] Dutta S. *et al.* Silver Nanoparticle Decorated Reduced Graphene Oxide (rGO) Nanosheet: A Platform for SERS Based Low-Level Detection of Uranyl Ion. ACS Applied Materials & Interfaces 5, 8724–8732 (2013). 23947790 10.1021/am4025017

[b37] Kwok D. Y. & Neumann A. W. Contact Angle Measurement and Contact Angle Interpretation. Adv. Colloid Interface Sci. 81, 167–249 (1999).

[b38] Wei N., Lv C. & Xu Z. Wetting of Graphene Oxide: A Molecular Dynamics Study. Langmuir 30, 3572–3578 (2014). 24611723 10.1021/la500513x

[b39] Hänni-Ciunel K., Findenegg G. H. & von Klitzing R. Water Contact Angle On Polyelectrolyte-Coated Surfaces: Effects of Film Swelling and Droplet Evaporation. Soft Mater. 5, 61–73 (2007).

[b40] Geens J., Van der Bruggen B. & Vandecasteele C. Characterisation of the Solvent Stability of Polymeric Nanofiltration Membranes by Measurement of Contact Angles and Swelling. Chem. Eng. Sci. 59, 1161–1164 (2004).

[b41] Han J. T. *et al.* Transparent Carbon Nanotube Patterns Templated by Inkjet-Printed Graphene Oxide Nanosheets. RSC Adv. 1, 44–47 (2011).

[b42] Chen D. *et al.* Impedance Study of Electrochemical Stability Limits for Electrolytes. Int. J. Electrochem. Sci. 7, 12383–12390 (2012).

[b43] Weingarth D. *et al.* Electrochemical Stability of Imidazolium Based Ionic Liquids Containing Cyano Groups in the Anion: A Cyclic Voltammetry, XPS and DFT study. J. Electrochem. Soc. 159, 611–615 (2012).

[b44] Ibrahim H. H. H. M. A., Mohammed S. S. A. E. R. & Amin A. Comparative Studies of the Electrochemical Behavior of Silver Electrode in Chloride, Bromide and Iodide Aqueous Solutions. Int. J. Electrochem. Sci. 5, 278–294 (2010).

[b45] Cheng T., Zhang Y., Lai W. Y. & Huang W. Stretchable Thin-Film Electrodes for Flexible Electronics with High Deformability and Stretchability. Adv. Mater. 27, 3349–3376 (2015). 25920067 10.1002/adma.201405864

[b46] Yang Z., Deng J., Sun X., Li H. & Peng H. Stretchable, Wearable Dye-Sensitized Solar Cells. Adv. Mater. 26, 2643–2647 (2014). 24648169 10.1002/adma.201400152

