# Supplementary material for: Impermeable flexible liquid barrier film for encapsulation of DSSC metal electrodes
Source: Sci Rep. 2016 Jun 6;6:27422. doi: 10.1038/srep27422 (PMC4893703; doi:10.1038/srep27422)
Supplement: Supplementary Information [file srep27422-s1.pdf]

Supporting information

Impermeable flexible liquid barrier film for encapsulation of DSSC metal electrodes

Junghee Yang[1], Misook Min[1, 2], Yeoheung Yoon[1], Wonjung Kim[3], Sol Kim[3], Hyoyoung Lee[1]*

[1]Centre for Integrated Nanostructure Physics (CINAP), Institute of Basic Science (IBS), Department of Chemistry, and Department of Energy Science, Sungkyunkwan University, 2066 Seobu-ro, Jangan-gu, Suwon-si, Gyeonggi-do, Korea.

[2]Current address: Department of Chemistry, Rice University, 6100 Main Street, Houston, Texas 77005

[3]Hyundai Motor Group, Environment & Energy Research Team, 37, Cheoldobangmulgwan-ro, Uiwang-si, Gyeonggi-do, Korea.

Supplementary Figures

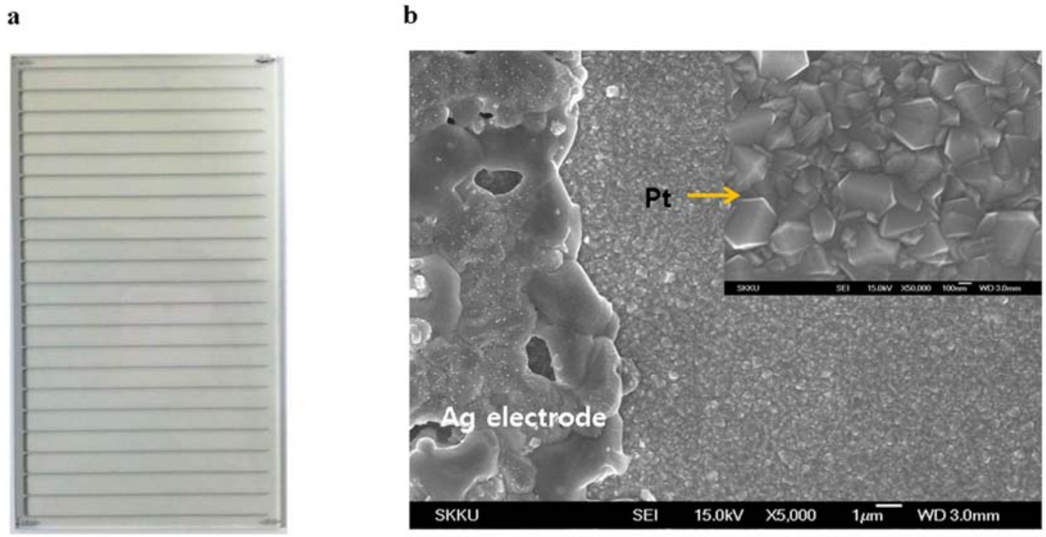

Supplementary Figure S1 (a) Counter electrode of parallel grid DSSC's substrate, (b) Top view, SEM image of Ag grid line with Pt used as a catalyst.

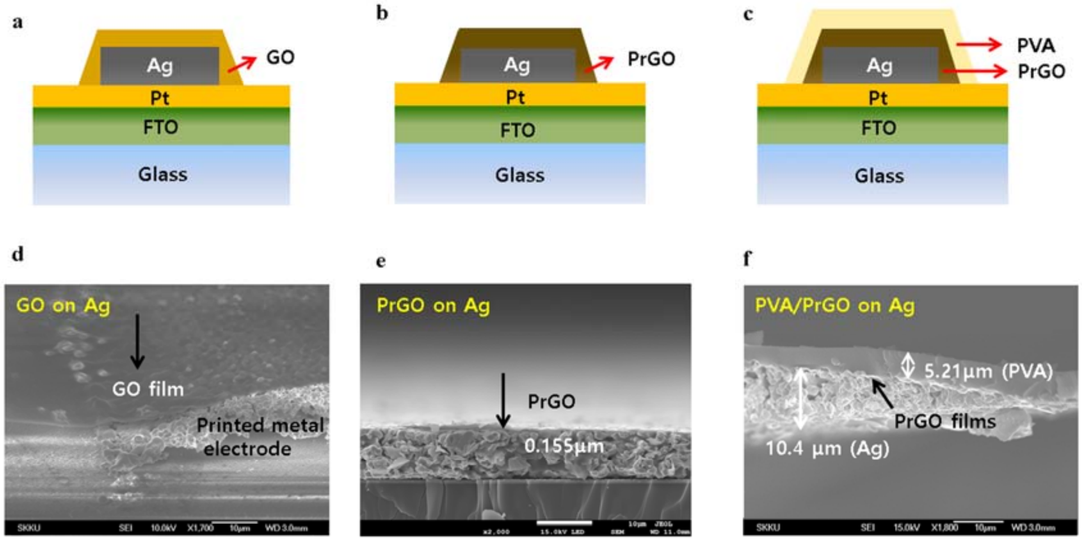

Supplementary Figure S2 Schematic procedures for fabrication of the PrGO/PVA film on Ag electrode, (a) GO, (b) PrGO, and (c) PVA/PrGO; SEM images of (d) GO, (e) PrGO, and (f) PVA/PrGO film on Ag electrodes.

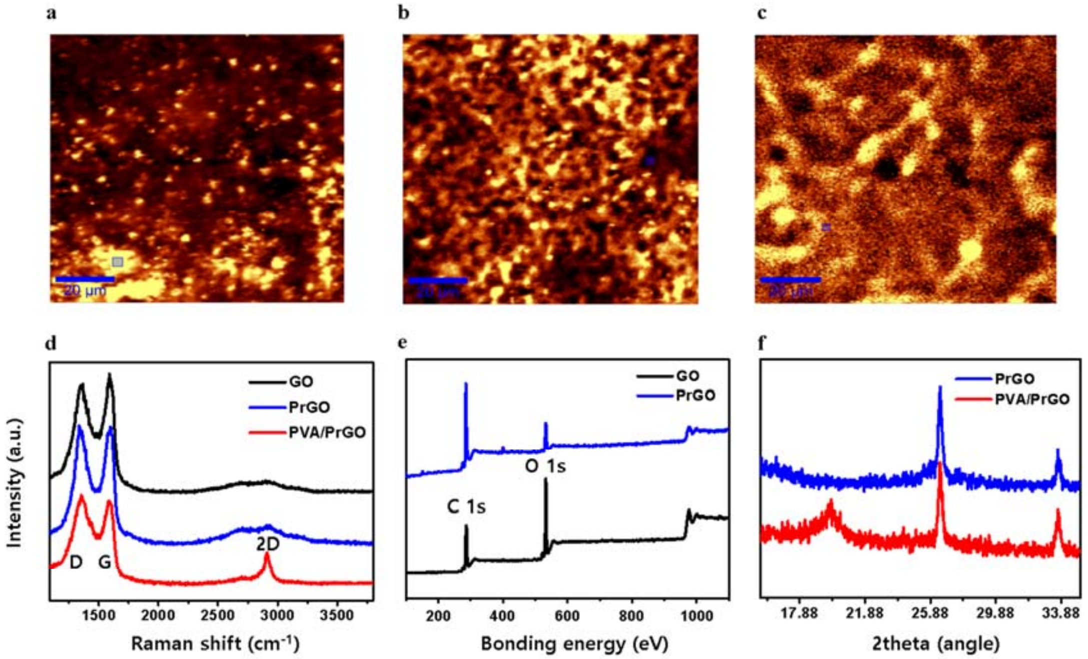

Supplementary Figure S3 Mapping Raman data in d band of (a) GO film, (b) PrGO film, (c) PVA/PrGO hybrid film, (d) especially Raman peak. (e) XPS data of GO and PrGO film, (f) XRD data of PrGO and PVA/PrGO film.

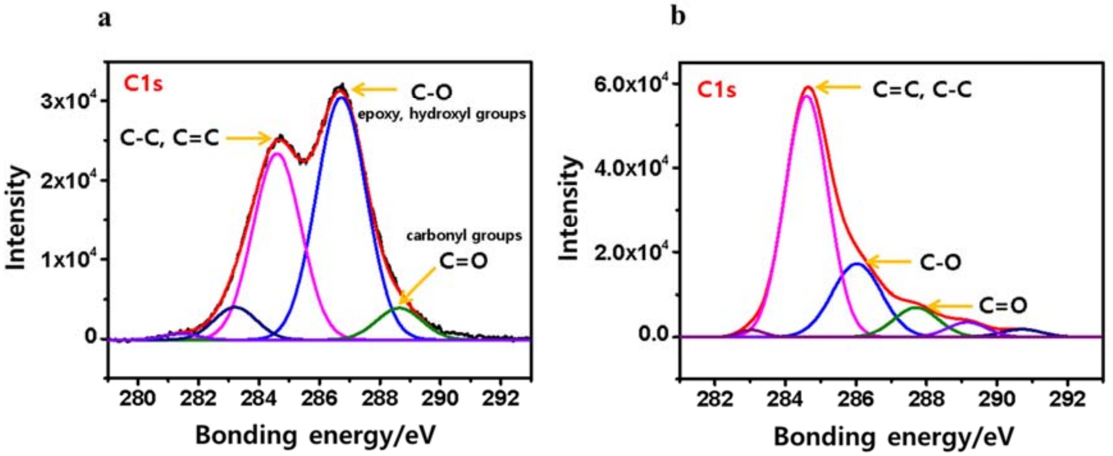

Supplementary Figure S4 (a) A typical XPS data of C1s in GO and (b) PrGO sheet.

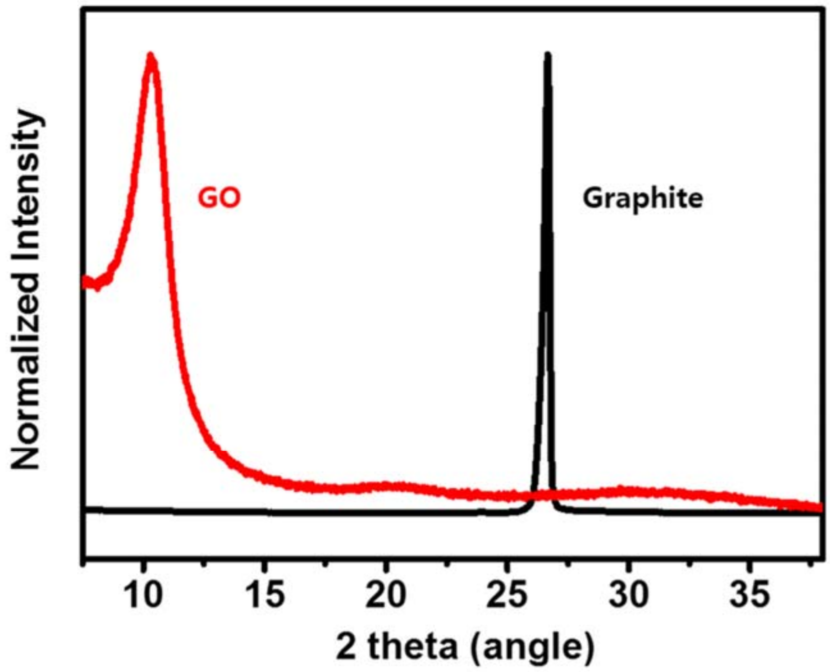

Supplementary Figure S5 XRD curve of GO by using chemical treatment and Graphite.

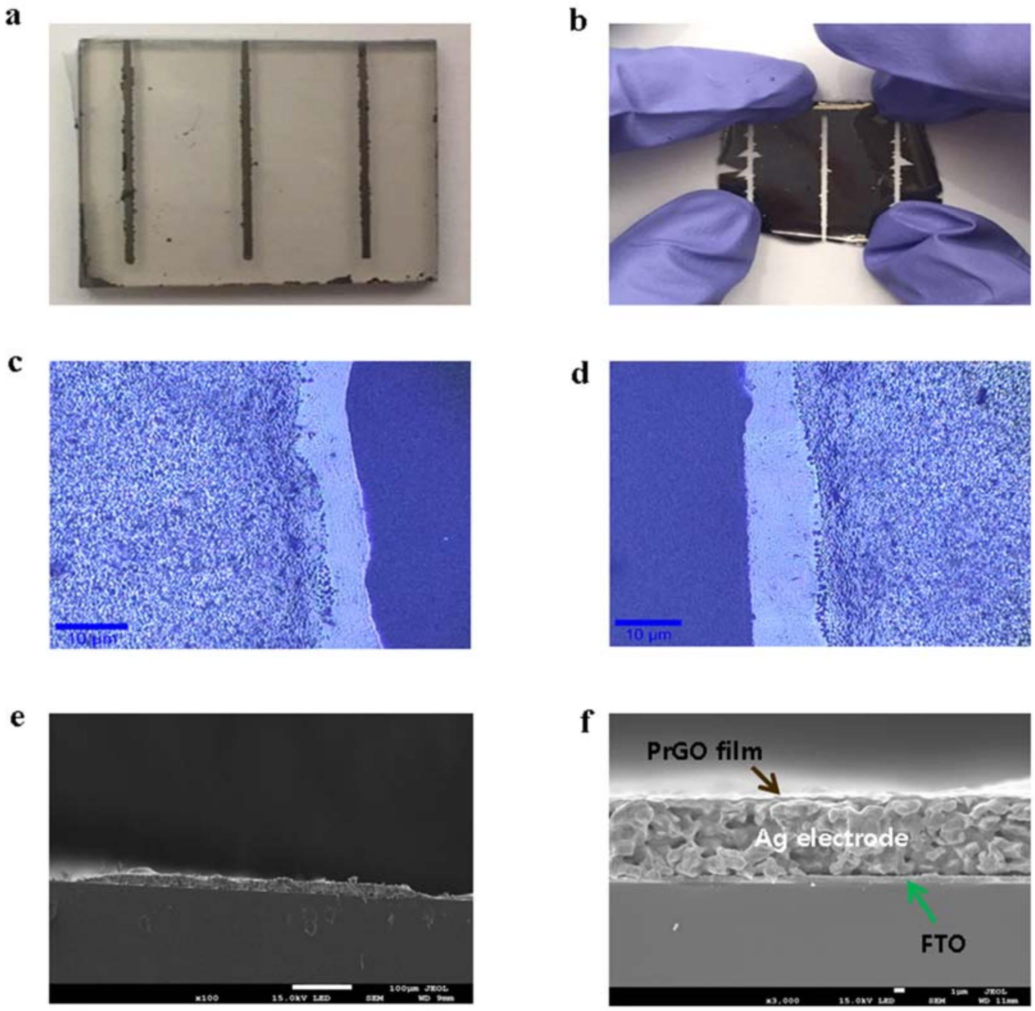

Supplementary Figure S6 The PrGO film that is attached to the Ag electrode on FTO glass show a good adhesion, which is confirmed with OM and SEM images. (a and b) Although the PrGO barrier film was tried to take off, the PrGO film on the Ag electrode was remained, (c and d) OM images, (e and f) SEM images.

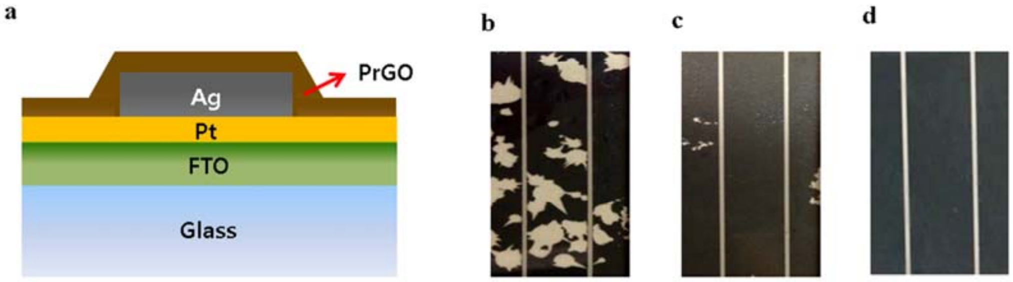

Supplementary Figure S7 PrGO coated substrates were prepared, (a) Schematic structure of PrGO/Ag electrode. Adhesion tests prepared at (b) 500 °C, (c) 400 °C, and (d) 300 °C in reduction temperature. The detachment test shows that the PrGO/Ag electrode prepared at 300 °C is the best preparation temperature.

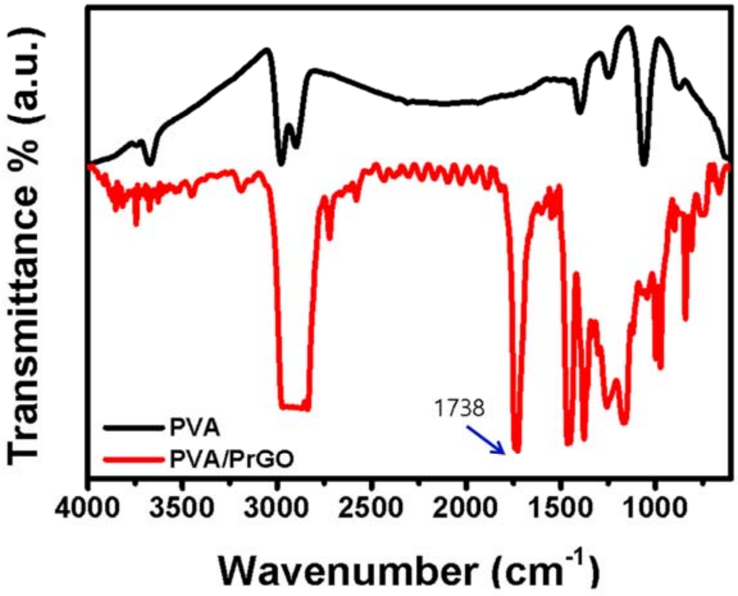

Supplementary Figure S8 FT-IR spectra of PVA and PVA/PrGO films, showing high intense C=O stretching peaks of ester functional group at 1738 cm^{-1} for the PVA/PrGO film.

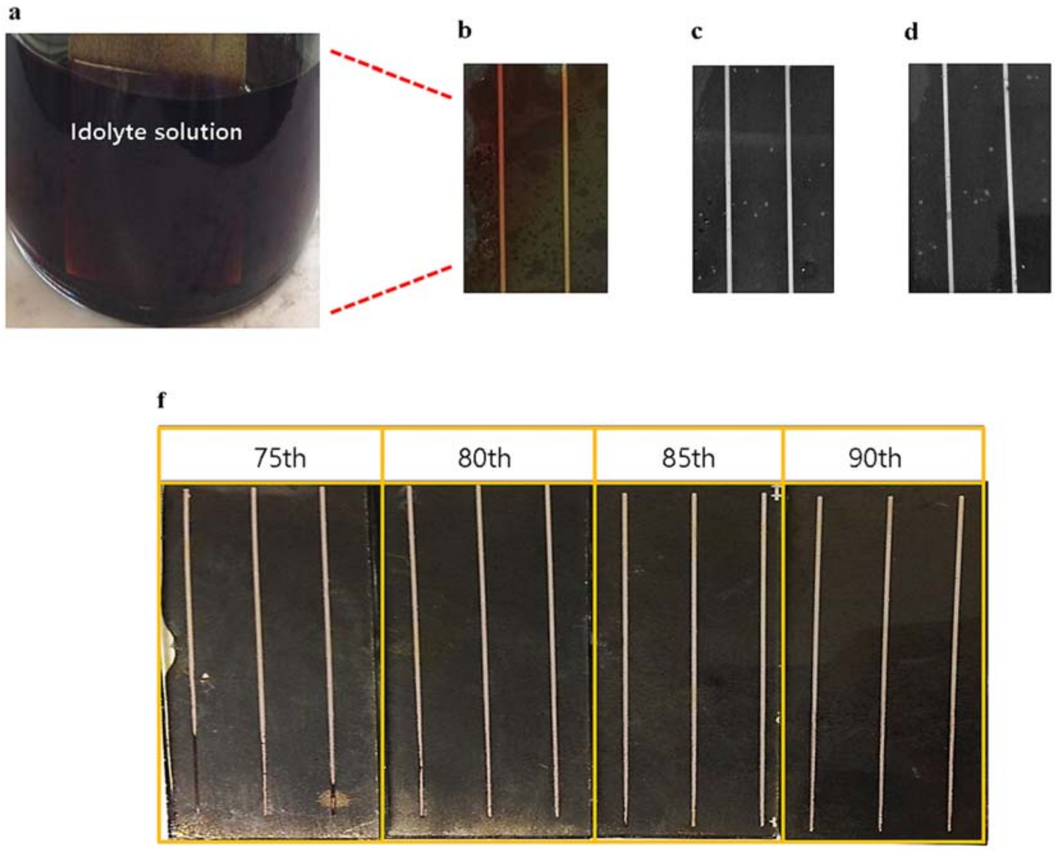

Supplementary Figure S9 (a) Photographs of PVA/PrGO/Ag electrode in iodolyte solution for electrolyte test, (b) Photograph of PVA/PrGO/Ag electrode immersed in iodolyte solution without washing the electrolyte, (c) After 200 hrs with washing the electrolyte, (d) After 500 hrs with washing the electrolyte. Although after 500 hrs, several black spots appeared by corrosion, the PVA/PrGO/Ag electrode was still endured for a long-term and (f) The number of GO solution coating and PVA coating (50th). After immersed 500 hrs, Ag electrode was corroded from edge.

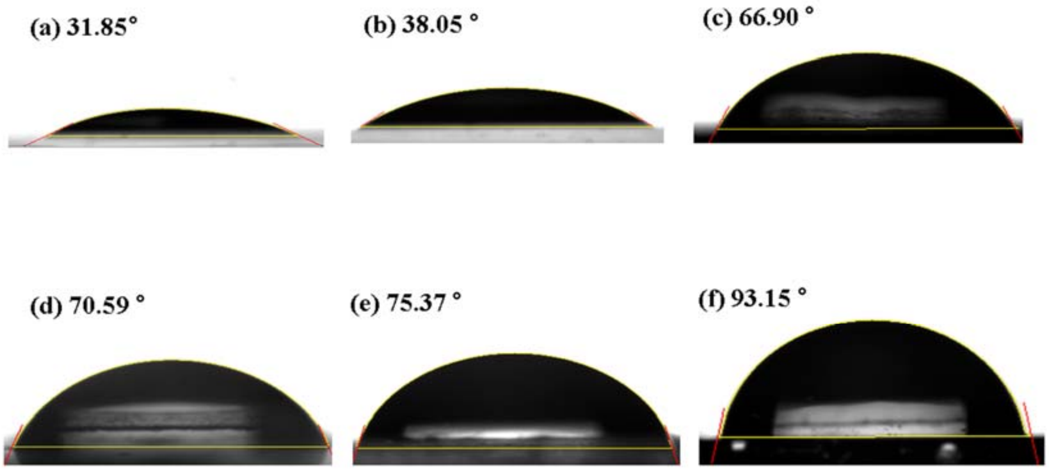

Supplementary Figure S10 (a) Water contact angle image of bare metal electrode on glass. (b) The images after coated GO. (c), (d), (e), and (f), WCA increases as the thickness of PrGO film increases, which means that the hydrophobicity increases as the thickness of PrGO film increases.

a

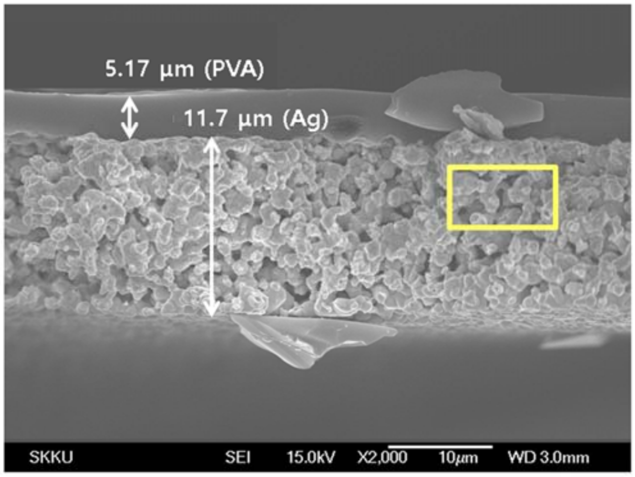

b

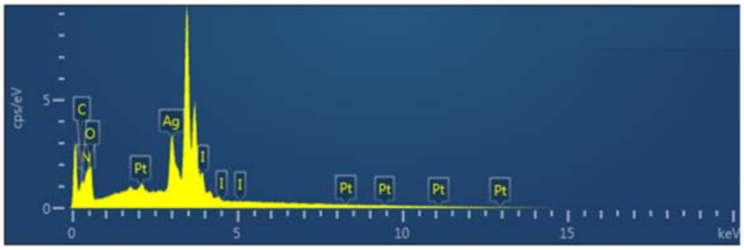

Element	Wt%
C	4.72
N	7.08
O	4.55
Ag	79.01
I	3.01
Pt	1.63

Supplementary Figure S11-1 EDX data of PVA/Ag (a) Cross section in SEM, (b) Existing elements after iodolyte electrolyte treatment. The iodolyte electrolyte solution is permeated through the film.

a

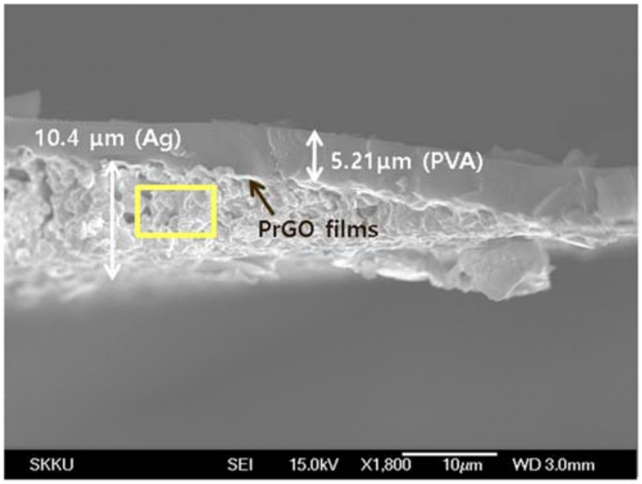

b

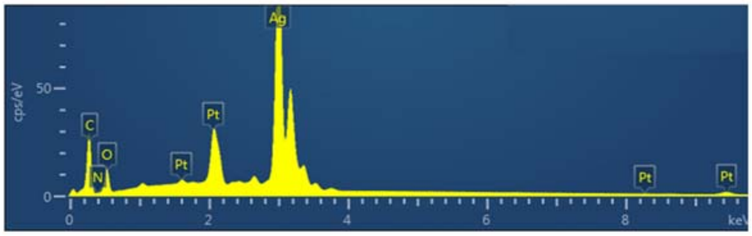

Element	Wt%
C	10.44
N	0.00
O	9.12
Ag	67.34
Pt	13.11

Supplementary Figure S11-2 EDX data of PVA-PrGO-Ag (a) Cross section in SEM, (b) Existing elements after iodolyte electrolyte treatment. The iodolyte electrolyte solution is not permeated through the PVA/PrGO film.

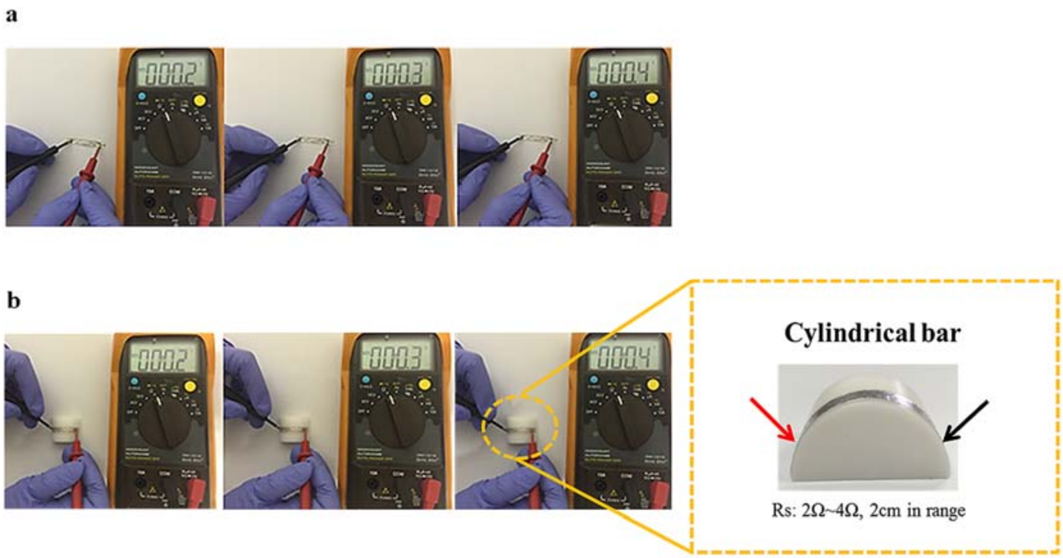

Supplementary Figure S12 Flexibility tests by measuring the change in sheet resistance of the PVA/PrGO/Ag electrode before and after bending on curved cylindrical bar. (a) Before bending, (b) After bending. The sheet resistances of the PVA/PrGO/Ag electrode were not changed, showing 2 **Ω** ~ 4 **Ω** sheet resistances.

Video 1 Adhesion test of PVA/PrGO/Ag film.
